# Evidence of orthohantavirus and leptospira infections in small mammals in an endemic area of Gampaha district in Sri Lanka

**DOI:** 10.1186/s42522-022-00073-y

**Published:** 2022-12-14

**Authors:** N. P. Sunil-Chandra, Åsa Fahlman, Shantha Waidyarathna, Jonas Näslund, M. V. M. L. Jayasundara, Lwande Olivia Wesula, Göran Bucht

**Affiliations:** 1 Department of Medical Microbiology, Faculty of Medicine, University of Kelaniya, Ragama, Sri Lanka; 2 Sri Lanka Institute of Biotechnology, Homagama, Sri Lanka; 3 Swedish Biodiversity Centre, Department of Rural and Urban Development, Faculty of Natural Resources and Agricultural Sciences, Swedish University of Agricultural Sciences, P.O. Box 7016, SE-750 07 Uppsala, Sweden; 4 Swedish Defence Research Agency, CBRN Defence and Security, Umeå, Sweden; 5 Department of Clinical Microbiology, Section for Virology, Umeå University, SE-901 85 Umeå, Sweden

**Keywords:** Sri Lanka, Orthohantavirus, Leptospira, Rodents, Shrews

## Abstract

**Background:**

Orthohantaviruses and leptospira are emerging zoonotic pathogens of high public health significance. The epidemiology of orthohantavirus infections and *leptospirosis* is similar and presents related clinical pictures in humans. However, a paucity of data on actual reservoir hosts for orthohantaviruses and leptospira exists. Therefore, this study aimed at determining the occurrence of orthohantaviruses and leptospira in small mammals captured in an endemic region of Sri Lanka.

**Methods:**

Rodents and shrews were morphologically and/or genetically identified using morphological keys and DNA barcoding techniques targeting the cytochrome oxidase b subunit gene (Cytb). Lung tissues and sera were subsequently analyzed for the presence of orthohantavirus RNA using qRT-PCR. Sera of rats were tested for IgG antibodies against orthohantaviruses and leptospira.

**Results:**

Forty-three (43) small mammals representing: *Rattus (R.) rattus* (black rat) or *R. tanezumi* (Asian rat), *Suncus murinus* (Asian house shrew), *R. norvegicus* (brown rat) and *Mus musculus* (house mouse) were investigated. No orthohantavirus RNA was detected from the lung tissue or serum samples of these animals. Elevated levels of IgG antibodies against Puumala orthohantavirus (PUUV) and/or Seoul orthohantavirus (SEOV) antigens were detected in sera of 28 (72%) out of the 39 rats analysed. Interestingly, 36 (92%) of the 39 rats also showed presence of anti leptospira-IgG antibodies in their serum, representing dual infection or dual exposure in 26/39 (66.7%) of examined rats.

**Conclusions:**

This project targets important public health questions concerning the occupational risk of orthohantavirus infections and/or leptospirosis in an endemic region of Sri Lanka. Most rats (72%) in our study displayed antibodies reacting to orthohantavirus NP antigens, related to PUUV and/or SEOV. No correlation between the orthohantavirus and leptospira IgG antibody levels were noticed. Finally, a combination of both morphological and DNA barcoding approaches revealed that several species of rats may play a role in the maintenance and transmission of orthohantavirus and leptospira in Sri Lanka*.*

**Supplementary Information:**

The online version contains supplementary material available at 10.1186/s42522-022-00073-y.

## Background

Orthohantaviruses and pathogenic spirochetes of genus *Leptospira* cause re-emerging global zoonoses clinically indistinguishable in human patients with similar epidemiology [1–6]. Rodents are important reservoirs for orthohantavirus and pathogenic leptospira [7].

Orthohantavirus infections in rodents are usually characterized by a transient viremia that peaks 7–14 days post infection, persistence of virus in lung, kidney, pancreas and spleen followed by a prolonged period when virus was rarely detected in blood, reflecting the effect of circulating neutralizing antibodies [8]. Virus infection induces a life-long IgG antibody response after 2–3 weeks of infection [9]. Horizontal transmission of orthohantavirus was shown in an experiment using laboratory rats at 35–63 days post inoculation, long after disappearance of virus in blood, oropharyngeal secretion, faeces and urine which may indicate that the virus shedding occurs intermittently or low titre viruria [8]. Another study reported that IgG serology is negative in the beginning of hantavirus infection, whereas PCR tests are positive [10]. However, the life-long persistence of hantaviruses in tissues and excreta is unclear due to the varying results obtained from previous studies on hantavirus pathogenesis and virus shedding in experimentally infected natural hosts with Hantaan, Puumala or Seoul orthohantavirus strains [8–15].

Orthohantaviruses are maintained by cyclical transmission between persistently infected rodents and humans may become infected through inhalation of aerosolized virus via contaminated urine, faeces and saliva, or from direct contact with animals [8, 15–18]. Orthohantavirus infections are asymptomatic in their rodent or insectivore natural hosts with which they have co-evolved [19] but the infection in humans cause a disease with varying morbidity and mortality rates depending on the orthohantavirus species and its geographic origin [20].

In contrast to orthohantaviruses, a wide variety of mammals may act as reservoirs for leptospira by harbouring the bacteria in their renal tubules. Rats (*Rattus* spp.) are the most important rodent reservoir and moreover the only rodent reservoir with a peridomestic presence worldwide, the other domestic and wild mammals may act as important maintenance or accidental hosts [21]. The infections occur mainly through direct or indirect contact with water sources contaminated with infected urine [22] as they are abundant in urban and peri-domestic environments. Presently, these pathogens have become a significant public health concern in tropical and temperate regions. A study on the sero-prevalence  of  leptospirosis in dairy cattle and peri-domestic rodents in Kandy district of Sri Lanka has shown that 20.3% of dairy cattle (reacting to serogroups Sejroe and Hebdomadis), 17.5% of peridomestic rodents (reacting to serogroups Javanica and Icterohaemorrhagiae), 20.3% of *B. bengalensis (Bandicota) and* 10.0% of *R. rattus* (rats) were seropositive to leptospirosis [10]. In another study, 10% of Cattle/buffaloes and 11% of rodents in the Gampaha district of Sri Lanka were found positive for leptospiral carriage by the real-time PCR indicating both cattle and rodents are important reservoirs for pathogenic leptospira species [12].

Orthohantaviruses are enveloped negative-sense single-stranded RNA viruses causing Hemorrhagic Fever with Renal Syndrome (HFRS) in Eurasia and orthohantavirus pulmonary syndrome (HPS) in the Americas [23]. Presently, the Orthohantaviru*s* genus in *Hantaviridae* family comprises 36 viruses (ICTV, Virus Taxonomy: 2018b Release). Until 2007, all discovered hantaviruses with the exception of the shrew-borne Thottapalayam virus [24], were detected in rodents (Mammalia, Rodentia). Since then, the recognized host range has expanded [25]. This far, only orthohantaviruses whose reservoirs are rodents (*Murinae, Arvicolinae and Sigmodontinae*) have been found to be pathogenic to humans [20].

Vitarana et al. reported orthohantavirus infections in rodents in Sri Lanka already in 1988. In their study, rats (*R. norvegicus*) captured in the Colombo Harbour area were reported seropositive for SEOV [26]. Presence of Thailand orthohantavirus infection in suspected leptospirosis patients in Kandy district was reported in 2011 [27]. Further, the presence of serum IgM antibodies reacting against PUUV and Hantaan orthohantavirus (HTNV) found in patients hospitalized with history of leptospirosis like-illness at the North Colombo Teaching Hospital in Gampaha district of Sri Lanka suggests either reactivity or cross-reactivity to both viruses [28]. In a more recent study, IgG antibodies to PUUV and/or HTNV strains were detected in patients with chronic kidney disease of unknown origin (CKDu) in North Central province of Sri Lanka, and the collective positivity of anti-orthohantavirus IgG antibodies was found significantly associated with the occurrence of CKDu [29].

Apart from two previous reports on orthohantavirus infection in rodents in Sri Lanka [1, 30], there are no studies carried out to identify rodent and other small mammal species harbouring orthohantavirus strains in Sri Lanka. Therefore, the identification of the rodent reservoir is of vital importance for the prevention and control of human hantavirus infections in Sri Lanka.

Enzyme Linked Immunosorbent Assay (ELISA) has been used to determine the sero-prevalence of leptospirosis in humans [28] and rodents [31]. More recently, another study concluded that ELISA is very useful for seroepidemiological purposes of past or present leptospiral infections in rodents [32].

Present study aimed at investigating the occurrence of orthohantavirus infection and leptospirosis in morphologically and/or genetically typed small mammals captured in an endemic region of Sri Lanka where human infections of orthohantavirus and leptospira had been diagnosed and reported previously sometimes presenting as a dual infection [28].

## Methods

### Sample collection

Blood and tissue samples were collected during March to September 2017 period from 43 free-ranging small mammals comprising of 39 rats, 1 mouse and 3 shrews inhabiting rural and urban areas in Gampaha district of the Western province of Sri Lanka. Shrews were morphologically identified whilst free ranging rodents were species identified morphologically and also by DNA barcoding techniques. This was followed by PCR screening of orthohantavirus RNA in lung tissues and sera from small mammals captured. Sera of 39 rats were tested additionally for the presence of IgG antibodies to PUUV and SEOV and leptospira genus specific antigens by ELISA but sera of three shrews (#14, #35 and #37) and one mouse (#22) were not tested.

**Fig. 1:**
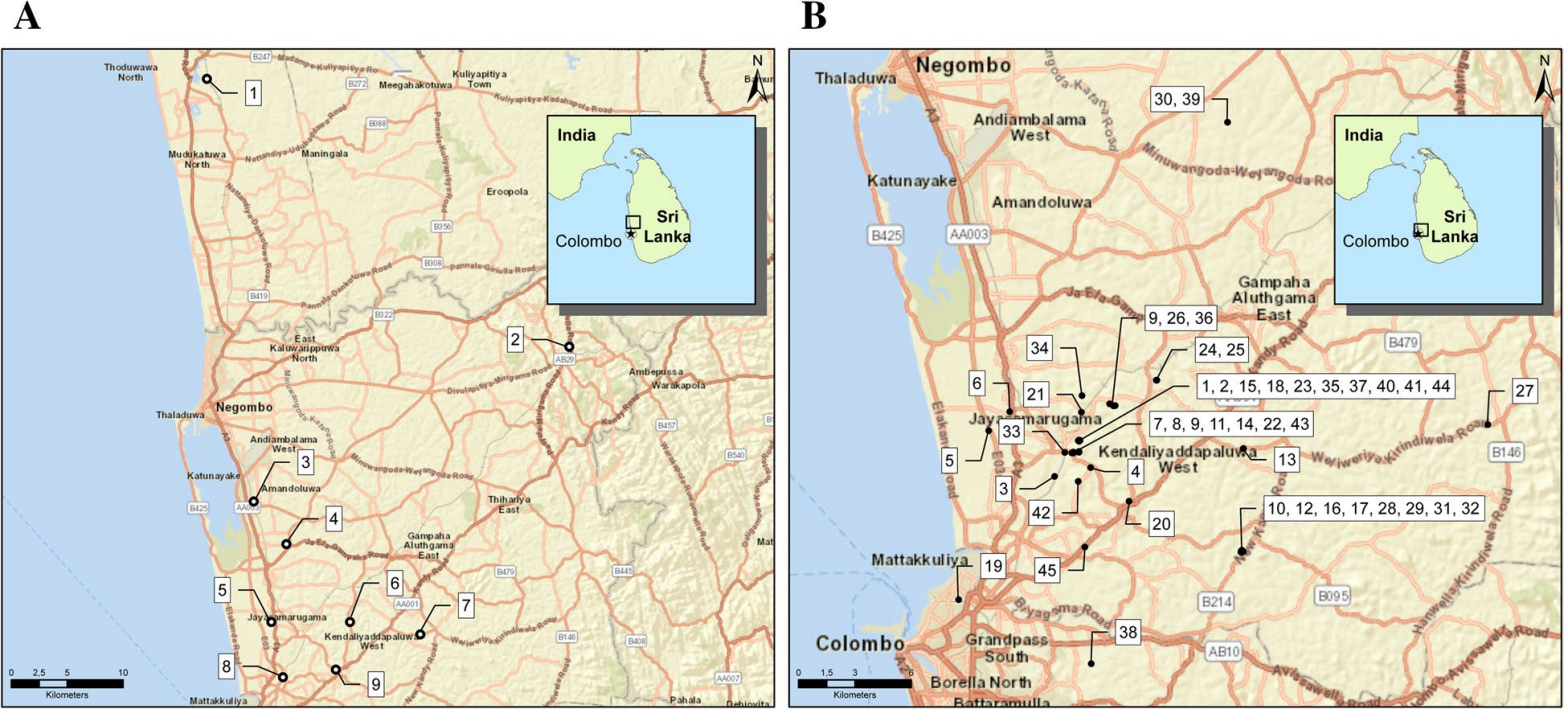
Maps demonstrating animal capture sites and human sampling locations. **A** shows the geographical position of the households where human samples were collected, and **B** shows the geographic locations where the rodents were captured. Sources used for displaying the sampling sites were: Esri, HERE, Garmin, USGS, Intermap, INCREMENT P, NRCan, Esri Japan, METI, Esri China (Hong Kong), Esri Korea, Esri (Thailand), NGCC,© OpenStreetMap contributors, and the GIS User Community Esri

Study area was chosen to involve locations from a previous study on hospitalized patients **(**Fig. 1A**)** with orthohantavirus-like illness and concomitant leptospirosis [28]. The geographic locations where the rodents and shrews were captured are shown in Fig. 1B.

Ten live-capture traps were deployed at or above ground level in the afternoon at different locations for trapping small mammals, and the study protocol was approved by the Department of Wildlife Conservation in Sri Lanka. Animal handling was carried out following human safety precautions with personal protection equipment (PPE). Live-trapped animals and their locations were recorded by geo-mapping. Euthanasia of live-trapped rodents was conducted after transportation to the laboratory according to The American Veterinary Medical Association (AVMA) guidelines for the euthanasia of animals: 2013 ed. 31 May 2015 [33]. The animals were photographed soon after euthanasia and dissected in a safety hood (category II) for collection of blood (heart puncture) and organs including lung, heart, kidney, liver, spleen, mesenteric lymph nodes, intestines and brain. Tissues and serum samples of 43 animals (40 rodents and 3 shrews) were stored at − 80 °C until analysis.

### Species identification of captured animals

Chromosomal DNA was extracted from lung tissues of the morphologically identified rodent species. Briefly, individual samples were homogenized with silica beads (1 mm) using MP FastPrep®-24 Instrument (MP Biomedicals, Santa Ana, CA, USA). Samples were thereafter digested with proteinase K (600 mAU/ ml) in buffer G2 (Qiagen Inc., Hilden, Germany) at 56 °C for 15 min under slow shaking, followed by a quick centrifugation to remove debris. Obtained supernatants were used for DNA extraction and isolation using Qiagen EZ1 Advanced Robotic Workstation (Qiagen Inc). Prior to PCR, primers were designed from available Cytb gene sequences of different local rodent species, see Table 1a.

**Table 1:** Primers

Primer name	Sequence	Melting point (Tm)
**(a). Cytochrome oxidase b subunit gene (Cytb) primers**		
Cytb 109F	CCC ATC CAA CAT CTC ATC ATG ATG A	61.3 °C
Cytb 183F	GCC TAT TCC TAG CAA TAC ACT ACA C	61.3 °C
Cytb 515F	ACC CTA GTC GAA TGA ATC TGA GG	60.6 °C
Cytb 267R	CCG TAG TTT ACG TCT CGG CAG AT	62.4 °C
Cytb 540R	CCT CCT CAG ATT CAT TCG ACT AG	60.6 °C
Cytb 669R	CCT GTG GGG TTR TTT GAT CCT GT	61.5 °C
**(b)** ***Orthohantavirus*** **primers**		
PanHanta-F1	ATG TAT GT [I] AGT GCW GAT GC	53.2 °C
PanHanta-R1	ACC A [I] TCW GW [I] CCA TCA YC	53.4 °C
PanHanta-F2	TGC WGA TGC [I] ACR AAA TGG TC	56.9 °C
PanHanta-R2	GCA TCA TCW GAR TGA TG [I] GCA A	57.5 °C
Seo242F	GAC AGG ATT GCA GCA GGG AAG A	62.1 °C
Seo870R	CAT CCC TGC AAG TGC ACC TTG	61.8 °C
Seo326F	CAC TAA GCT ATG GGA ATA CAC TGG A	61.3 °C
Seo1077R	ATG AGG AAC ACA ATC ATG GCT TCA A	59.7 °C

Briefly, nine mitochondrial sequences were downloaded from GenBank and aligned by ClustalW of BioEdit package version 7.1.3.0 [34]. Conserved regions between different rodent sequences were identified and primers within Cytb gene were constructed thereof.

PCR reactions were conducted with different combinations of Cytb primers and extracted DNA samples in standard 96-well plates using KAPA SYBR® FAST qPCR Kit (KAPA Biosystems, Boston, MA, USA) along with CFX96™ 143 Real-time detection system (Bio-Rad Laboratories, Hercules, CA, USA). PCR reaction was carried out in 25 μl mixtures containing 1 μl DNA template, 25 μM of each primer and 12.5 μl of KAPA master mix. Initial denaturation at 95 °C for 3 min was followed by 40 cycles, each consisting of 5 s at 95 °C; 20 s at 55 or 57 °C and 15 s at 72 °C. Finally, the samples were heated to 95 °C for 10 s, cooled down to 65 °C and re-heated from 65 °C to 90 °C at a rate of 0.5 °C per 5 s.

PCR products were analysed by agarose electrophoresis and purified with illustra MicroSpin S-400 HR Columns™ (GE Healthcare, Sweden) before sequencing the amplicons of samples (Eurofins MWG Operon, Ebersberg, Germany) using amplification primers. Sequence comparisons were finally conducted with corresponding sequences in databases by Standard Nucleotide BLAST analysis.

### Detection of orthohantavirus RNA

Total RNA from serum samples or lung tissues were extracted with TRIzol® LS Reagent (Invitrogen™ Life technologies) in a total volume of 1 ml before RNA isolation using RNeasy® Mini Kit and RNeasy® Columns as recommended by the manufacturer (Qiagen, Hilden, Germany). RNA was recovered in 20 μl nuclease-free water. Obtained RNA was stored at − 80 °C before complementary deoxyribonucleic acid (cDNA) synthesis with GoScript Reverse Transcriptase Kit (Promega, Madison, WI, USA) was performed. RT-PCR was carried out using the procedures summarized below; KAPA SYBR® FAST qPCR Kit (KAPA Biosystems, Boston, MA, USA) and SsoFastTM EvaGreen® Supermix (Bio-Rad, Hercules, CA, USA) Kit along with orthohantavirus primers purchased from MWG-Biotech AG (Ebersberg, Germany) were used, see Table 1b.

The basic conditions for PCR kits were those recommended by the manufacturer. Deoxyinosine (dI) was used at sites in the Pan-Hanta primers where more than two different bases are found by a multiple alignment of thirty-three L-segment sequences, representing a wide variety of different orthohantaviruses*.* Accurate annealing temperatures for primer pairs were investigated individually by temperature gradients starting at 45 °C and ending at 55 °C. RT-PCR was carried out in 25 μl mixtures containing 1 μl cDNA template, 25 μM of each primer and 12.5 μl of KAPA master mix (KAPA SYBR® FAST qPCR Kit, KAPA Biosystems, Boston, MA, USA). An initial denaturation step at 95 °C for 3 min was followed after a total of 35 cycles, each consisting of a denaturation step at 95 °C for 15 s and an annealing temperature of 56 °C for 15 s [35].

### Antigen purification

DNA constructs encoding the amino-terminal part of the nucleocapsid (N) protein of PUUV (AY526219) and SEOV (M34881) were expressed from poly-histidine-fusion vectors in *Escherichia coli BL*-21 DE3 (Invitrogen™ Life technologies). The N proteins were purified using metal chelating chromatography according to a protocol from QIAexpressionist 01/2000 (Qiagen Ltd., UK) [36].

### Detection of rat anti- orthohantavirus antibodies

Indirect ELISA was performed on sera collected from 39 rats to determine IgG antibodies to N proteins of PUUV and SEOV. Analysis was essentially done as described earlier [36]. Calculated cut-off value was set to 3 times the optical density (OD) value of negative sera derived from serologically negative laboratory rats (*R. norvegicus*). Briefly, microtiter plates (Nunc MaxiSorpTM) were coated overnight at 4 °C with 50 μl containing truncated forms of the N proteins (1–3 μg/ ml) in ELISA-coating buffer (carbonate-bicarbonate buffer pH 9.2 to 10.6). Plates were blocked thereafter with 10 mg/ ml of Casein Blocking Buffer (antibodies-online, cat no: ABIN929980) and incubated at 37 °C for 2 hours. Plates were thereafter washed four times with phosphate buffered saline (PBS) containing 0.05% Tween, pipetted 50 μl rat sera diluted 100x in Casein Blocking Buffer supplemented with 0.05% Tween-20® (MERCK, Schuchardt, Germany), incubated for one hour at room temperature. Plates were thereafter washed four times with PBS containing 0.05% Tween, pipetted 50 μl of a secondary horseradish peroxidase (HRP) conjugated goat anti-rat immunoglobulin (H + L) (Invitrogen™ Life technologies), diluted according to the manufacturer’s instructions and incubated 1 h at room temperature. Plates were then washed eight times and finally 100 μl of substrate 3,3′,5,5′- Tetramethylbenzidine (TMB) (Seramun Diagnostica, Heidesee Germany) was added to each well and incubated 15 minutes at room temperature. Reaction was stopped by addition of 0.25 M H_2_SO_4_; (100 μl/ well) and OD at 450 nm was determined. Two separate OD measurements with duplicates of each serum were analysed [see Additional file 1].

### Detection of rat anti-leptospira IgG

ELISA for quantitative in vitro determination of Rat IgG antibodies to Genus specific leptospira antigens (Creative Diagnostics, USA) was performed on sera from 39 rats and results were interpreted according to manufacturer’s protocol. Sera which gave > 23.5 pg/ ml were considered positive and undetectable levels are below < 23.5 pg/ ml according to manufacturer’s instructions.

Briefly, 50 μl of 1 in 5 diluted rat serum was pipetted to each well of microtiter plate pre-coated with Genus specific leptospira antigens except the blank well and incubated 30 minutes at 37 °C. Thereafter, emptied wells, dried by swing, added 300 μl washing buffer to each well, stilled for 30 s, drained, repeated 5 times and dried by pat. Each well was pipetted 50 μl of ready to use anti-Rat IgG HRP conjugate except the blank well, incubated 30 minutes at 37 °C and washed six times. Then added 50 μl TMB Chromogen Solution A and 50 μl TMB Chromogen Solution B to each well, mixed gently, incubated 15 minutes at 37 °C evading light. Added stop solution (50 μl/ well) to each well and read OD at 450 nm within 15 minutes using a Biotek-Ex800 ELISA reader. Concentration of anti-leptospira IgG in rat serum was determined by comparing the OD of the sample with the standard curve derived from ODs to a range of concentrations (150 pg/ ml to 1800 pg/ ml) of the standard reagent (purified rat leptospira IgG) that binds with Genus specific leptospira antigen pre-coated microtitre plate wells in the Rat leptospira IgG ELISA, and the additional figure file shows this in more detail [see Additional file 2].

Research permission and approval to study rodent-borne infectious agents in Sri Lanka was granted by the Department of Wildlife Conservation in Sri Lanka subject to the provisions of Fauna and Flora Protection Ordinance (FFPO) of Sri Lanka (Ref: WL/3/2/85/17) and animal tissues were obtained solely for the purpose of this study.

## Results

A total of 43 individual small mammals captured between March and September 2017 were morphologically identified and photographed before lung tissues were prepared for genetic species determination by sequencing a short barcoding region of mitochondrial cytb gene. Of the 43 captured animals, 25 were identified both by barcoding and morphology as rats; 24 *R. rattus* (also called black rat, or roof rat) and *R. tanezumi*, (also called Asian rat, or Asian house rat) and one (1) rodent was identified as the brown rat (*R. norvegicus*). Three (3) animals were morphologically identified as Asian house shrews (*Suncus murinus*), and one (1) animal as house mouse (*Mus musculus*). Fourteen (14) rodents were not identified genetically, but instead morphologically as *R. rattus*/ *R. tanezumi*.

Lung and serum samples were successively screened for orthohantavirus RNA and for antibodies against PUUV and SEOV orthohantavirus N proteins, respectively, by in-house methods [37, 38]. With comparison to internal controls, the negative results obtained from RT-PCR of captured rodents strongly indicate that orthohantavirus RNA was not present in the samples extracted from lung or serum samples of collected small mammals.

Our serological data indicated that 28 (72%) of the 39 rats showed elevated IgG antibody titres against PUUV and/ or SEOV orthohantavirus N proteins, see Table 2 and Additional file 1**.**

**Table 2:** Individual cross-reactivity of rat sera to orthohantavirus antigens

Specificity and positivity	Individual rat identification No. (#)	Number of rats tested for anti-orthohantavirus IgG
Positive SEOV & negative PUUV	24, 42	28 / 39 rats tested positive for anti-orthohantavirus IgG antibodies. 18/28 rats were positive for IgG against both PUUV and SEOV orthohantavirus N protein antigen specificities. 7 /28 rats were positive for IgG against PUUV orthohantavirus N protein antigen specificity only 3 /28 rats were positive for IgG against SEOV orthohantaviru*s* N protein antigen specificity only
Positive SEOV & positive PUUV	2, 43*	
Positive PUUV & negative SEOV	3, 4, 5**, 15, 25**, 31	
Strong positive PUUV & positive SEOV	6*, 13*, 23, 29*, 38*, 44*	
Strong positive SEOV & strong positive PUUV	7, 17*, 27, 28*, 33***, 41*	
Strong positive SEOV & positive PUUV	16, 20*, 36, 39*	
Strong positive SEOV & negative PUUV	18	
Strong positive PUUV & negative SEOV	21*	
Negative SEOV & negative PUUV	8*, 9*, 10*, 11*, 12*, 19*, 26*, 30*, 32*, 34*, 40	11/39 rats were negative for anti-orthohantavirus IgG antibodies.

**Note**: Species Identified by bar coding include R rattus*, *R tanezumi* ** and R norvegicus***. Other rats were morphologically identified

**Fig. 2:**
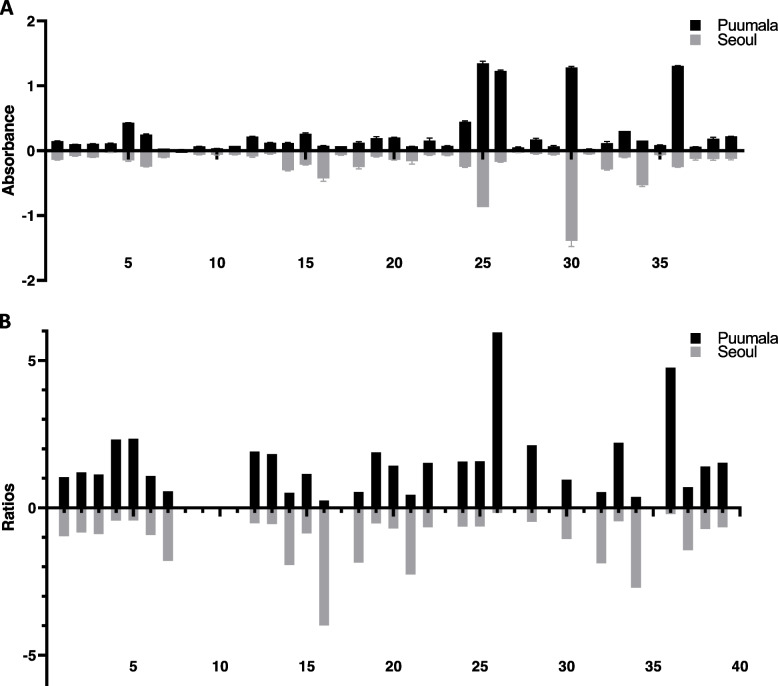
The black bars in Fig. 2A show the mean ELISA OD values of positive rodent (rat) sera as calculated from two separate ELISA measurements and double samples of each when using the PUUV N protein as antigen. The corresponding gray bars pointing downwards indicate the mean ELISA OD values when using the SEOV N-protein antigen with the same set of samples. The bars in Fig. 2B show the absorbance ratios. The upper black bars in Fig. 2B indicate the ratios calculated from mean OD values from PUUV N-protein antigen divided by the corresponding OD values when using the SEOV N-protein -antigen. The gray bars in Fig. 2B indicate the inverse ratios calculated from mean OD values from SEOV N-protein antigen divided by the corresponding OD values when using the PUUV N-protein antigen. Negative and positive control rat sera were included in the assay. Sera of 3 shrews (#14, #35 and #37) and one mouse (#22) were not tested for orthohantavirus IgG due to unavailability of anti-shrew or anti-mouse IgG detector antibodies

Of those, we detected antibodies targeting both PUUV and SEOV N proteins in serum samples of 18 rats. Sera of 7/28 and 3/28 rats captured in Sri Lanka reacted only to PUUV N protein or SEOV N protein respectively. Interestingly, rodent #28 and #33 (*R. rattus* and *R. norvegicus, respectively*) showed remarkable OD-values against both PUUV and SEOV N proteins as noticed by ELISA measurements, see Fig. 2A. On the other hand, some rats such as *R. rattus* #29 and #41 showed higher reactivity against the PUUV N protein than against the SEOV N protein. The ratios in the reactivity against the two orthohantavirus N proteins are shown in Fig. 2B. Other rodent sera such as #18, #20, #24, #36 and #39 showed the reverse ELISA reactivity. The ratios of the OD values were stronger against the SEOV N protein than towards the PUUV N protein. Nearly similar observations were found in an earlier report which used human sera collected in the same region [28].

**Fig. 3:**
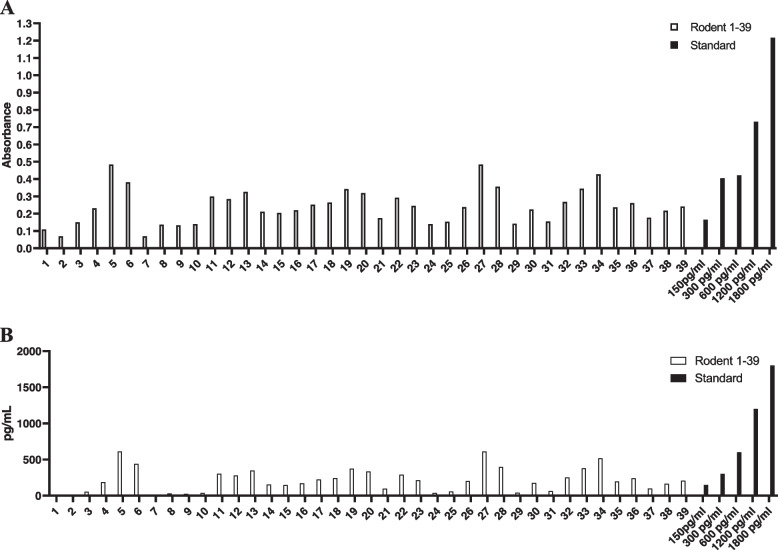
These two graphs show the mean ELISA OD values (**A**) and the individual quantity of rat leptospira IgG (pg/mL) (**B**) of 39 rat sera as calculated from the standard curve plotted according to manufacturer’s protocol (Creative Diagnostics, USA). Sera of 3 shrews (#14, #35 and #37) and one mouse (#22) were not tested for leptospira IgG due to unavailability of anti-shrew or anti-mouse IgG detector antibodies

According to the data from Rat leptospira IgG ELISA, it was a surprise to find that only 3 rats (#2, #3 and #8) of the 39 rats fell under the detection limit of the leptospira assay (23.5 pg/ ml). Remaining 36 rats (92%) including genetically identified R. rattus*, *R tanezumi* ** and *R. norvegicus* showed leptospira IgG levels in the higher range of the standard curve, see Table 3, Fig. 3, and Additional files 1 and 2. Sera of three shrews (#14, #35 and #37) and one mouse (#22) were not tested for leptospira IgG due to unavailability of anti-shrew or anti-mouse IgG detector antibodies. However, no correlation between the presence of leptospira IgG and orthohantavirus IgG levels was noticed for the collected individual animal samples.

**Table 3:** Individual levels of rat anti-leptospira IgG

Anti-leptospira rat IgG concentration range at 1/5 dilution of sera	Anti-leptospira IgG detectable (+) and below detection limit (−)	Individual rat identification No. (#)	Number of rats tested
< 23.5 pg/mL	–	2, 3, 8*,	3
23.5 pg/mL -150 pg/mL	+	4, 9*, 10*, 11*, 17*, 24, 27, 28*, 32*, 34*, 42	11
> 150 pg/ml -300 pg/mL	++	43*, 44*, 41*, 40, 36, 33***, 29*, 26*, 25**, 20*, 19*, 18, 16, 13*, 5**	15
> 300 pg/ml -600 pg/mL	+++	7, 12*, 15, 21*, 23, 31, 38*, 39*	8
> 600 pg/ml - 1200 pg/mL	++++	6*, 30*	02
> 1200 pg/ml -1800 pg/mL	+++++		None

**Note:** Species Identified by bar coding include R rattus*, *R tanezumi* ** and R norvegicus***. Other rats were morphologically identified

## Discussion

PUUV is transmitted by the bank vole (*Myodes glareolus*). Bank vole has an exclusively Palearctic distribution with a range across Europe and North-Western Asia. Consequently, our serological data regarding the strong reactivity to the PUUV N protein antigen reflect cross-reactivity to a still unidentified orthohantavirus in Sri Lanka. The higher degree of infection by arvicolid PUUV, often found in a murid reservoir (rats) is confusing, and suggests a cross-reaction with hither to unknown novel hantaviruses. Interestingly, in neighboring South India, having almost the same small mammal fauna as in Sri-Lanka, serious and even fatal HFRS cases, remarkably also mimicking leptospirosis, were described as being caused by a “PUUV-like agent” [39].

However, the serological reactivity to the SEOV N protein antigen was less surprising. Rats are natural vectors for SEOV, and their geographic distribution is worldwide. Of the 43 captured small mammals, 25 were identified both by barcoding and morphology as rats. Another 14 rats, 3 Asian house shrews and a house mouse (*Mus musculus*) were morphologically identified. A few questions arise from these data; does our serological data suggest that two orthohantaviruses are independently circulating in Sri Lanka, or are these serological findings due to individual cross-reactivity among the rodents to the two orthohantavirus antigens used? In the present study, detection of IgG antibodies to orthohantavirus or leptospira were performed in rats only. Therefore, it is important that further studies should be carried out to detect antibodies to orthohantaviruses and leptospira in shrews and mice by ELISA using anti-shrew and anti-mouse IgG - HRP conjugates respectively.

In most cases, SEOV antibodies show a stronger cross-reactivity to Dobrava, Saaremaa orthohantavirus (DOBV/SAAV) and HNTV N proteins, while PUUV antibodies show a higher degree of cross-relativity to Tula (TULV) and orthohantaviruses N proteins of the New World such as Sin Nombre orthohantavirus (SINV) N protein [28]. SEOV and PUUV belong to different cross-reacting serogroups, perhaps due to the distant relation between rodent vectors, and between different sero-groups, the cross-reactivity is normally weak and sometimes absent [40–42]. However, in the 1980s, both for humans and rodents, relied only on these cross-reactions, since only the (murid) prototype HTNV was used for detecting what now appears to be mainly PUUV-infections.

Large efforts were taken to detect orthohantavirus RNA from the lung tissue or serum samples of small mammals. However, earlier findings from Singapore [43] have established an orthohantavirus seroprevalence of about 30% there. In contrast, a comparable Pan-hanta RT-PCR performed on samples of *Rattus norvegicus* and *Rattus tanezumi* in Singapore indicated that only about 2% of the animals were PCR positive. The detection of virus RNA was attempted to demonstrate the presence and the hantavirus strain (s) of orthohantaviruses circulating in region of interest. However, none of the rodents (rats) tested were positive indicating that the rodents did not have an active infection or did not carry the virus asymptomatically during the time of trapping.

Absence of orthohantavirus RNA from extracts of blood and lung tissues of captured small mammals in the present study may have caused due to the neutralizing effect of anti-orthohantavirus IgG in seropositive animals, or absence/ undetectable levels of viral RNA at the time of capture as reported previously [16, 17, 21, 44]. Furthermore, the absence [16, 17, 44] as well as presence [17, 19, 45] of viral RNA or virus in blood and or lung tissues of seropositive rodent hosts has been previously reported in several studies which is also reviewed by Meyer and Schmaljohn [15].

In this present study, no attempts were taken to detect DNA, or isolate pathogenic Leptospira spp. from the animals. Clearly, the presence of orthohantavirus RNA or leptospira antigen (or DNA) is only possible to detect during acute infection. In contrast, serological evidence can be detected in rodents even after acute infection.

Our findings reveal different genetically identified rat species *R. rattus* (also called black rat, or roof rat), *R. tanezumi*, (also called Asian rat, or Asian house rat) and *R. norvegicus* (brown rat) contribute to the circulation and transmission of novel orthohantavirus pathogens in Sri Lanka (Figs. 2 & 3, Table 2).

High prevalence of leptospira in the captured animals indicates a widespread distribution of the pathogen in the Gampaha district and perhaps elsewhere in Sri Lanka. The fact that most rodents screened in this study, except three individuals #2, #3, #8, were positive for IgG antibodies against leptospira suggests their role as important reservoirs of leptospira which is consistent with previous studies carried out in other endemic localities in Sri Lanka [46]. In our study, *R. rattus and/or R. tanezumi, R. norvegicus* were found to have antibodies against Leptospira.

## Conclusions

This study shows that several morphologically and genetically identified rat species captured in Gampaha district have serological evidence to past infections with orthohantaviruses and or leptospira*,* and possibly responsible for human infections in the region. *R. rattus* is most likely involved in the transmission of both pathogens. Therefore, genetic identification of the rodents in the present study confirms that *Rattus rattus, Rattus tanezumi* and *Rattus norvegicus* contribute to the circulation and transmission of novel orthohantavirus pathogens in Sri Lanka.

Also, findings of the present study on the evidence to hantavirus infection and leptospirosis among genetically identified local rodents is a continuation and confirmation of a former observation in humans [28], namely acute patients hospitalized in Sri Lanka suffering from concomitant leptospirosis and hantavirus infection. Since both human infections are highly similar (thrombocytopenia with acute kidney & liver injury), and since the omnipresent wild urban rat is confirmed once again, this time in Sri Lanka, as the prime source and reservoir of double infection, implications for a potentially worldwide underestimated problem because  human leptospirosis can be cured by antibiotics, but acute hantavirus infection cannot.

Therefore, the findings point to the likelihood of concomitant infections as previously reported in human patients [28] and or sequential infections with both orthohantaviruses and Leptospira in rodents. Additional studies linking seroepidemiology and epizootiology are needed to better understand and determine the burden of orthohantavirus infections and leptospirosis, as well the animal reservoirs.

## Supplementary Information

**Additional file 1. **Individual ELISA reactivity to SEOV and PUUV N-protein antigens. The Table file shows the reactivity of individual rat sera against SEOV and PUUV N- protein antigens with OD values of two separate ELISA measurements in duplicate samples of each and the results of morphological and genetic identification of captured small mammals. Keys to anti- Puumala or anti-Seoul rat IgG ELISA positivity in rat sera, anti-*leptospira* rat IgG ELISA positivity in rat sera and morphological and genetic identification of small mammals are given in the foot notes.

**Additional file 2.** The standard curve to determine the amount of leptospira antibodies in sera of captured animals (rats). The graph shows the mean optical density (OD) to a range of concentrations (150 pg/mL to 1800 pg/mL) of standard reagent (purified rat leptospira IgG) that binds with Genus specific leptospira antigen pre-coated microtiter plate wells in the Rat leptospira IgG ELISA.

## Data Availability

Sequencing data generated in this study are available from GenBank (accession MW152252-MW152275).

## Abbreviations

IgG: Immunoglobulin G
DNA: Deoxyribonucleic acid
Cytb: Cytochrome oxidase b subunit gene
RNA: Ribonucleic acid
qRT-PCR: Quantitative Real-time Polymerase chain reaction
PUUV: Puumala orthohantavirus
SEOV: Seoul orthohantavirus antigens
N: nucleocapsid
HFRS: Hemorrhagic Fever with Renal Syndrome
HPS: Hantavirus Pulmonary Syndrome
ICTV: International Committee on Taxonomy of Viruses
HTNV: Hantaan orthohantavirus
CKDu: Chronic kidney disease of unknown origin
ELISA: Enzyme Linked Immunosorbent Assay
PPE: Personal protection equipment
AVMA: American Veterinary Medical Association
cDNA: Complementary deoxyribonucleic acid
dI: Deoxyinosine
OD: Optical density
PBS: Phosphate buffered saline
HRP: Horseradish peroxidase
TMB: 3,3′,5,5′- Tetramethylbenzidine
FFPO: Fauna and Flora Protection Ordinance
DOBV/SAAV: Dobrava, Saaremaa orthohantavirus
TULV: Tula virus
SINV: Sin Nombre orthohantavirus
