# Supplementary material for: Evidence of orthohantavirus and leptospira infections in small mammals in an endemic area of Gampaha district in Sri Lanka
Source: One Health Outlook. 2022 Dec 14;4:17. doi: 10.1186/s42522-022-00073-y (PMC9749280; doi:10.1186/s42522-022-00073-y)
Supplement: Supplementary file 1 — Additional file 1. Individual ELISA reactivity to SEOV and PUUV N-protein antigens. The Table file shows the reactivity of individual rat sera against SEOV and PUUV N- protein antigens with OD values of two separate ELISA measurements in duplicate samples of each and the results of morphological and genetic identification of captured small mammals. Keys to anti- Puumala or anti-Seoul rat IgG ELISA positivity in rat sera, anti-leptospira rat IgG ELISA positivity in rat sera and morphological and genetic identification of small mammals are given in the foot notes. [file 42522_2022_73_MOESM1_ESM.docx]

**Additional file 1:**

**Title: Individual ELISA reactivity to SEOV and PUUV N-protein antigens**

**Description:**

The Table file shows the reactivity of individual rat sera against SEOV and PUUV N- protein antigens with OD values of two separate ELISA measurements in duplicate samples of each and the results of morphological and genetic identification of captured small mammals. Keys to anti- Puumala or anti-Seoul rat IgG ELISA positivity in rat sera, anti-*leptospira* rat IgG ELISA positivity in rat sera and morphological and genetic identification of small mammals are given in the foot notes

| ***Species barcoding*** | **Serum no.** | **ELISA -1 – PUUV OD value** | | **ELISA -2 – PUUV OD value** | | **ELISA-1 –SEOV**  **OD value** | | **ELISA-2 –SEOV**  **OD value** | | **Sum PUUV**  **(+/-,D)** | **Sum**  **SEOV**  **(+/-,D)** | **PUUV/SEOV**  **(P/S/PS)** | **Anti-Lepto Rat IgG**  **ELISA** | **Dual exposure**  **(IgG**  **positive**  **for both Hanta &**  **Lepto)** |
| --- | --- | --- | --- | --- | --- | --- | --- | --- | --- | --- | --- | --- | --- | --- |
|  |  | **Well1** | **Well2** | **Well1** | **Well2** | **Well1** | **Well2** | **Well1** | **Well2** |  |  |  |  |  |
| ***R rattus/R tanezumi*** | 02 | 0.162 | 0.067 | 0.150 | 0.144 | 0.148 | 0.075 | 0.137 | 0.142 | 0.523 (P+) | 0.502 (S+) | 1.04 H+  (P+S+) | **L (-)** | **H+** |
| ***R rattus/R tanezumi*** | 03 | 0.126 | 0.063 | 0.101 | 0.099 | 0.106 | 0.055 | 0.084 | 0.079 | 0.389  (p+) | 0.324  (S-) | 1.2H+  (P+S-) | **L (-)** | **H+** |
| ***R rattus/R tanezumi*** | 04 | 0.162 | 0.072 | 0.107 | 0.105 | 0.131 | 0.059 | 0.105 | 0.101 | 0.446  (P+) | 0.396  (S-) | 1.1H+  (P+S-) | **L (+)** | **H+L+** |
| ***Rattus tanezumi*** | 05 | 0.116 | 0.059 | 0.108 | 0.116 | 0.065 | 0.052 | 0.028 | 0.027 | **0.399**  **(P+)** | 0.172  (S-) | 2.31H+  (P+S-) | **L (++)** | **H+L+** |
| ***Rattus rattus*** | 06 | 0.270 | 0.111 | 0.434 | 0.428 | 0.166 | 0.068 | 0.158 | 0.138 | 1.243  (P++) | 0.530  (S+) | 2.345 H++  (P++S+) | **L (++++)** | **H+L+** |
| ***R rattus/R tanezumi*** | 07 | 0.216 | 0.079 | 0.243 | 0.257 | 0.175 | 0.069 | 0.249 | 0.242 | 0.795  (P++) | 0.735  (S++) | 1.08H++  (P++S++) | **L (+++)** | **H+L+** |
| ***Rattus rattus*** | 08 | 0.093 | 0.057 | 0.034 | 0.033 | 0.121 | 0.061 | 0.108 | 0.101 | 0.217  (P-) | 0.391  (S-) | 0.554H-  (P-S-) | **L (-)** | **H-L-** |
| ***Rattus rattus*** | 09 | 0.05 | 0.048 | 0.016 | 0.009 | 0.05 | 0.052 | 0.015 | 0.015 | 0.123  (P-) | 0.132  (S-) | 0.93H-  (P-S-) | **L (+)** | **L+** |
| ***Rattus rattus*** | 10 | 0.104 | 0.072 | 0.069 | 0.067 | 0.097 | 0.060 | 0.064 | 0.062 | 0.312  (P-) | 0.283  (S-) | 1.10H-  (P-H-) | **L (+)** | **L+** |
| ***Rattus rattus*** | 11 | 0.075 | 0.054 | 0.036 | 0.035 | 0.081 | 0.052 | 0.064 | 0.065 | 0.200  (P-) | 0.262  (S-) | 0.76H-  (P-S-) | **L (+)** | **L+** |
| ***Rattus rattus*** | 12 | 0.114 | 0.055 | 0.074 | 0.074 | 0.097 | 0.054 | 0.062 | 0.064 | 0.317  (P-) | 0.277  (S-) | 1.14H-  (P-S-) | **L (+++)** | **L+** |
| ***Rattus rattus*** | 13 | 0.231 | 0.072 | 0.214 | 0.221 | 0.147 | 0.063 | 0.098 | 0.079 | 0.738  (P++) | 0.387  (S-) | 1.9H++  (P++S-) | **L (++)** | **H+L+** |
| ***Suncus murinus*** | 14 | NT | NT | NT | NT | NT | NT | NT | NT | NT | NT | NT | **NT** | **NT** |
| ***R rattus/R tanezumi*** | 15 | 0.123 | 0.061 | 0.127 | 0.121 | 0.087 | 0.053 | 0.052 | 0.045 | 0.432  (P+) | 0.237  (S-) | 1.82H+  (P+S-) | **L (+++)** | **H+L+** |
| ***R rattus/R tanezumi*** | 16 | 0.145 | 0.064 | 0.116 | 0.127 | 0.200 | 0.079 | 0.307 | 0.293 | 0.452  (P+) | 0.879  (S++) | 0.514H++  (P+S++) | **L (++)** | **H+L+** |
| ***Rattus rattus*** | 17 | 0.217 | 0.081 | 0.251 | 0.271 | 0.220 | 0.070 | 0.206 | 0.218 | 0.820  (P++) | 0.714  (S++) | 1.15H++  (P++S++) | **L (+)** | **H+L+** |
| ***R rattus/R tanezumi*** | 18 | 0.104 | 0.056 | 0.071 | 0.079 | 0.269 | 0.113 | 0.458 | 0.397 | 0.310  (P-) | 1.237  (S++) | 0.25H++  (P-S++) | **L (++)** | **H+L+** |
| ***Rattus rattus*** | 19 | 0.109 | 0.056 | 0.07 | 0.07 | 0.09 | 0.052 | 0.07 | 0.061 | 0.305  (P-) | 0.273  (S-) | 1.11H-  (P-S-) | **L (++)** | **L+** |
| ***Rattus rattus*** | 20 | 0.093 | 0.061 | 0.111 | 0.133 | 0.154 | 0.089 | 0.271 | 0.227 | 0.398  (P+ | 0.741  (S++) | 0.53H++  (P+/-S++) | **L (++)** | **H+L+** |
| ***Rattus rattus*** | 21 | 0.171 | 0.078 | 0.174 | 0.210 | 0.100 | 0.056 | 0.095 | 0.085 | 0.633  (P++) | 0.336  (S-) | 1.88H++  (P++S-) | **L (+++)** | **H+L+** |
| ***Mus musculus*** | 22 | NT | NT | NT | NT | NT | NT | NT | NT | NT | NT | NT | **NT** | **NT** |
| ***R rattus/R tanezumi*** | 23 | 0.196 | 0.085 | 0.207 | 0.200 | 0.134 | 0.067 | 0.142 | 0.139 | 0.688  (P++) | 0.482  (S+) | 1.43H++  (P++S+) | **L (+++)** | **H+L+** |
| ***R rattus/R tanezumi*** | 24 | 0.048 | 0.048 | 0.064 | 0.068 | 0.123 | 0.067 | 0.133 | 0.192 | 0.228  (P-) | 0.515  (S+) | 0.443H+  (P-S+) | **L (+)** | **H+L+** |
| ***Rattus tanezumi*** | 25 | 0.051 | 0.049 | 0.131 | 0.184 | 0.084 | 0.055 | 0.063 | 0.071 | 0.415  (P+) | 0.273  (S_) | 1.52H+  (P+S-) | **L (++)** | **H+L+** |
| ***Rattus rattus*** | 26 | 0.111 | 0.061 | 0.07 | 0.076 | 0.098 | 0.06 | 0.067 | 0.074 | 0.318  (P-) | 0.299  (S-) | 1.063H-  (P-S-) | **L (++)** | **L+** |
| ***R rattus/R tanezumi*** | 27 | 0.247 | 0.095 | 0.439 | 0.456 | 0.219 | 0.073 | 0.254 | 0.243 | 1.237  (P++) | 0.789  (S++) | 1.567H++  (P++S++) | **L (+)** | **H+L+** |
| ***Rattus rattus*** | 28 | 0.612 | 0.38 | 1.369 | 1.323 | 0.449 | 0.15 | 0.868 | 0.868 | 3.684  (P++) | 2.335  (S++) | 1.578H++  (P++S++) | **L (+)** | **H+L+** |
| ***Rattus rattus*** | 29 | 0.566 | 0.363 | 1.218 | 1.241 | 0.166 | 0.063 | 0.175 | 0.165 | 3.388  (P++) | 0.569  (S+) | 5.95H++  (P++S+) | **L (++)** | **H+L+** |
| ***Rattus rattus*** | 30 | 0.074 | 0.06 | 0.046 | 0.056 | 0.059 | 0.051 | 0.028 | 0.031 | 0.236  (P-) | 0.169  (S-) | 1.396H-  (P-S-) | **L (++++)** | **L+** |
| ***R rattus/R tanezumi*** | 31 | 0.100 | 0.059 | 0.159 | 0.186 | 0.08 | 0.056 | 0.05 | 0.052 | 0.504  (P+) | 0.238  (S-) | 2.117H+  (P+S-) | **L (+++)** | **H+L+** |
| ***Rattus rattus*** | 32 | 0.081 | 0.055 | 0.06 | 0.078 | 0.081 | 0.057 | 0.066 | 0.061 | 0.274  (P-) | 0.265  (S-) | 1.03H-  (P-S-) | **L (+)** | **L+** |
| ***Rattus norvegicus*** | 33 | 0.516 | 0.362 | 1.272 | 1.295 | 0.560 | 0.288 | 1.328 | 1.450 | 3.445  (P++) | 3.626  (S++) | 0.95 H++  (P++S++) | **L (++)** | **H+L+** |
| ***Rattus rattus*** | 34 | 0.069 | 0.055 | 0.025 | 0.027 | 0.082 | 0.054 | 0.055 | 0.050 | 0.176  (P-) | 0.241  (S-) | 0.73H-  (P-S-) | **L (+)** | **L+** |
| ***Suncus murinus*** | 35 | NT | NT | NT | NT | NT | NT | NT | NT | NT | NT | NT | **NT** | **NT** |
| ***R rattus/R tanezumi*** | 36 | 0.142 | 0.07 | 0.098 | 0.135 | 0.177 | 0.08 | 0.294 | 0.283 | 0.445  (P+) | 0.834  (S++) | 0.533H++  (P+S++) | **L (++)** | **H+L+** |
| ***Suncus murinus*** | 37 | NT | NT | NT | NT | NT | NT | NT | NT | NT | NT | NT | **NT** | **NT** |
| ***Rattus rattus*** | 38 | 0.219 | 0.077 | 0.305 | 0.305 | 0.144 | 0.059 | 0.106 | 0.101 | 0.906  (P++) | 0.410  (S+) | 2.21(H++  (P++S+) | **L (+++)** | **H+L+** |
| ***Rattus rattus*** | 39 | 0.146 | 0.069 | 0.157 | 0.158 | 0.264 | 0.112 | 0.545 | 0.515 | 0.530  (P+) | 1.436  (S++) | 0.369H++  (P+ S++) | **L (+++)** | **H+L+** |
| ***R rattus/R tanezumi*** | 40 | 0.097 | 0.059 | 0.089 | 0.084 | 0.088 | 0.062 | 0.062 | 0.068 | 0.329  (P-) | 0.280  (S-) | 1.175H-  (P-S-) | **L (++)** | **L+** |
| ***Rattus rattus*** | 41 | 0.630 | 0.443 | 1.305 | 1.310 | 0.182 | 0.091 | 0.249 | 0.253 | 3.688  (P++) | 0.775  (S++) | 4.758H++  (P++S++) | **L (++)** | **H+L+** |
| ***R rattus/R tanezumi*** | 42 | 0.088 | 0.081 | 0.062 | 0.060 | 0.109 | 0.063 | 0.139 | 0.107 | 0.291  (P-) | 0.418  (S+) | 0.696H+  (P-S+) | **L (+)** | **H+L+** |
| ***Rattus rattus*** | 43 | 0.136 | 0.071 | 0.202 | 0.171 | 0.096 | 0.062 | 0.143 | 0.113 | 0.580  (P+) | 0.414  (S+) | 1.401H+  (P+S+) | **L (++)** | **H+L+** |
| ***Rattus rattus*** | 44 | 0.127 | 0.077 | 0.219 | 0.223 | 0.107 | 0.068 | 0.136 | 0.111 | 0.646  (P++) | 0.422  (S+) | 1.53H++  (P++S+) | **L (++)** | **H+L+** |

|  |  | |  |  |  |  |  |  |  |  |  |  |  |  | |
| --- | --- | --- | --- | --- | --- | --- | --- | --- | --- | --- | --- | --- | --- | --- | --- |
|  |  | |  |  |  |  |  |  |  |  |  |  |  |  | |
|  | +C | | 0.214 | 0.156 | 0.392 | 0.423 | 0.099 | 0.063 | 0.127 | 0.104 | 1.185 **(P++)** | 0.393  **(S+/-)** | **3.01 (P++)** |  | |
|  | -C1 | | 0.049 | 0.049 | 0.015 | 0.014 | 0.051 | 0.049 | 0.016 | 0.017 | 0.127 | 0.133 |  |  | |
|  | -C2 | | 0.053 | 0.050 | 0.012 | 0.014 | 0.050 | 0.054 | 0.015 | 0.016 | 0.129 | 0.135 |  |  | |
|  | -C3 | | 0.052 | 0.050 | 0.012 | 0.018 | 0.050 | 0.051 | 0.016 | 0.018 | 0.132 | 0.135 |  |  | |
|  | -C4 | | 0.051 | 0.054 | 0.008 | 0.010 | 0.049 | 0.051 | 0.020 | 0.023 | 0.123 | 0.143 |  |  | |
|  |  | |  |  |  |  |  |  |  | Mean Sum PUUV –ve control  (mean C1-C4)  0.127+  0.129+  0.132+  0.123 = **0.511**  0.511/4 **= 0.128** | | Mean Sum SEOV  –ve control (mean C1-C4)  0.133+  0.135+  0.135+  0.143 = **0.546**  0.546/4 **= 0.136** | |  | |
|  |  | |  |  |  |  |  |  |  | ***0.128x3* =0.383**  **+** | | ***0.136x3* = 0.408**  **+** | |  | |
|  |  | |  |  |  |  |  |  |  | ***0.128x5* = 0.639**  **PUU++** | | ***0.136x5* = 0.680**  **SE0++** | |  | |
| **Key 1: Anti- Puumala or anti Seoul rat IgG ELISA positivity in rodent sera:**  Strong positive (++) = x 5 above the negative value  Weak positive (+) = x3 above but below to x5 the negative value  Negative (-) = Below the x3 negative  D = doubtful,  NT = Not tested | | | | | | | | | | | | | | |  |
|  | | Strongly positive for Hantavirus IgG (strong positivity to PUU & weak positivity to SEO) | | | | | | | | | | | | |  |
|  | | Strongly positive for Hantavirus IgG (strong positivity to PUU & negative SEO) | | | | | | | | | | | | |  |
|  | | Positive for Hantavirus IgG (positive PUU and negative SEO) | | | | | | | | | | | | |  |
|  | | Positive for Hantavirus IgG (positive SEO and positive PUU) | | | | | | | | | | | | |  |
|  | | Strongly positive for Hantavirus IgG (strong positivity to SEO & weak positivity to PUU) | | | | | | | | | | | | |  |
|  | | Strongly positive for Hantavirus IgG (strong positivity to SEO & negative to PUU | | | | | | | | | | | | |  |
|  | | Negative for Hantavirus IgG (negative PUU and negative SEO) | | | | | | | | | | | | |  |
|  | | Strongly positive for Hantavirus IgG (strong positivity to SEO & strong positivity to PUU) | | | | | | | | | | | | |  |
|  | | Positive for Hantavirus IgG (positive SEO and negative PUU) | | | | | | | | | | | | |  |

| **Key 2: Anti-*leptospira* rat IgG ELISA positivity in small mammals sera**  **(Anti-*Leptospira* rat IgG concentration pg/ml rodent sera)**  Undetectable  <23.5pg/ml: (L-)  Detectable  23.5pg/ml -150pg/ml (L+)  >150pg/ml -300pg/ml (L++)  >300pg/ml -600pg/ml (L+++)  >600pg/ml - 1200pg/ml (L++++) |
| --- |

| **Key 3: Small mammals** | | | |
| --- | --- | --- | --- |
| **Species** | | **Morphologically identified** | **Genetically identified** |
|  | ***Rattus tanezumi*** *(Asian house rat)* | ***Yes*** | ***Yes*** |
|  | ***Rattus rattus/ tanezumi*** | ***Yes*** | *No* |
|  | ***Rattus rattus*** (black rat) | ***Yes*** | ***Yes*** |
|  | ***Mus musculus*** | ***Yes*** | *No* |
|  | ***Rattus norvegicus*** (brown rat) | ***Yes*** | ***Yes*** |
|  | ***Suncus murinus*** (Asian house shrew) | ***Yes*** | *No* |
