# Supplementary material for: Evidence of orthohantavirus and leptospira infections in small mammals in an endemic area of Gampaha district in Sri Lanka
Source: One Health Outlook. 2022 Dec 14;4:17. doi: 10.1186/s42522-022-00073-y (PMC9749280; doi:10.1186/s42522-022-00073-y)
Supplement: Supplementary file 2 — Additional file 2. The standard curve to determine the amount of leptospira antibodies in sera of captured animals (rats). The graph shows the mean optical density (OD) to a range of concentrations (150 pg/mL to 1800 pg/mL) of standard reagent (purified rat leptospira IgG) that binds with Genus specific leptospira antigen pre-coated microtiter plate wells in the Rat leptospira IgG ELISA. [file 42522_2022_73_MOESM2_ESM.docx]

**Additional file 2:**

| **Concentration of standards (pg/ml)** | **Mean OD** |
| --- | --- |
| 150 | 0.1645 |
| 300 | 0.4040 |
| 600 | 0.4215 |
| 1200 | 0.7315 |
| 1800 | 1.2165 |

**Title**: The standard curve to determine the amount of *Leptospira* antibodies in sera of captured animals (rats).

**Description:**

The graph shows the mean optical density (OD) to a range of concentrations (150pg/mL to 1800pg/mL) of standard reagent (purified rat *Leptospira* IgG) that binds with Genus specific *Leptospira* antigen pre-coated microtiter plate wells in the Rat *Leptospira* IgG ELISA.
